# Draft Genome Sequence of *Microdochium bolleyi*, a Dark Septate Fungal Endophyte of Beach Grass

**DOI:** 10.1128/genomeA.00270-16

**Published:** 2016-04-28

**Authors:** Aaron S. David, Sajeet Haridas, Kurt LaButti, Joanne Lim, Anna Lipzen, Mei Wang, Kerrie Barry, Igor V. Grigoriev, Joseph W. Spatafora, Georgiana May

**Affiliations:** a Department of Ecology, Evolution and Behavior, University of Minnesota, Saint Paul, Minnesota, USA; b U.S. Department of Energy Joint Genome Institute, Walnut Creek, California, USA; c Department of Botany and Plant Pathology, Oregon State University, Corvallis, Oregon, USA; d Department of Plant Biology, University of Minnesota, Saint Paul, Minnesota, USA

## Abstract

Here, we present the genome sequence of the dark septate fungal endophyte *Microdochium bolleyi* (*Ascomycota, Sordariomycetes, Xylariales*). The assembled genome size was 38.84 Mbp and consisted of 173 scaffolds and 13,177 predicted genes.

## GENOME ANNOUNCEMENT

*Microdochium bolleyi* (syn.: *Idriella bolleyi*) is a fungus commonly found growing endophytically within plant roots, particularly those of grasses (1). It is characterized as a dark septate endophyte due to its melanized cell walls and intra- and intercellular growth within the roots of healthy plants (2). In culture, *M. bolleyi* produces one-celled, crescent-shaped conidia (3). Its hyphae are dark brown and may release an orange pigment (3). *M. bolleyi* frequently associates with native and invasive beach grasses on coastal dunes of the U.S. Pacific Northwest (4). These dunes present harsh environments for plant and fungal growth due to high winds and salt spray and low soil moisture and nutrients (5, 6), making them important ecosystems for understanding fungal diversity. We sequenced the genome of a strain of *M. bolleyi* (J235TASD1), isolated from surface-sterilized roots of *Ammophila breviligulata* in Pacific City, Oregon, USA. To our knowledge, only one previous study has sequenced the genome of *M. bolleyi* (7), and ours is the first publicly available genome.

We identified J235TASD1 as *M. bolleyi* based on high similarity of the ITS region to sequences of references strains CBS137.64 (GenBank accession no. AM502264) and CBS172.63 (AM502265) (8), and to sequences (AJ279454, AJ279475) identical to that from a culture identified using morphological characteristics (9). Prior to DNA and RNA extractions, the fungus was grown in 2% malt extract liquid media at room temperature. We extracted DNA using the Qiagen DNEasy plant minikit (Valencia, CA, USA). RNA was extracted using a Trizol and chloroform protocol, and purified using the Ambion PureLink RNA minikit (Austin, TX, USA). Sequencing and annotation followed the U.S. Department of Energy Joint Genome Institute (JGI) pipeline (10). Genomic 2 × 150-bp reads from a single 300-bp insert library were obtained using Illumina HiSeq2500 and initially assembled using Velvet (11). The resulting assembly was used to simulate long 3-kb mate pairs that were then assembled together with the original reads using AllPathsLG version R49403 (12) and annotated using the JGI Annotation pipeline (10). The transcriptome was *de novo* assembled using Rnnotator version 3.4.0 (13).

The assembled genome was 38.84 Mbp and consisted of 215 contigs and 173 scaffolds. Sequencing read depth coverage was 136.2×. The assembled transcriptome consisted of 18,493 consensus contigs, of which 99% mapped to the genome assembly to confirm its completeness. Annotation resulted in 13,177 gene models. Median gene length was 1,516 bp and median protein length was 377 amino acids. The estimated haploid genome size was 40.14 Mbp with an estimated genome repeat of 4.0% (25-bp *k*-mer). The J235TASD1 genome is larger than the *M. bolleyi* genome reported by Jewell (7) (38.16-Mbp genome, 13,047 predicted genes, 8,060 annotated genes). The genome of our *M. bolleyi* strain may help illuminate how fungi tolerate stressful environmental conditions.

### Nucleotide sequence accession numbers.

The genome sequences and annotations are available from the JGI fungal genome portal MycoCosm (10). This whole-genome shotgun project has been deposited at DDBJ/EMBL/GenBank under the accession number LSSP00000000. The version described in this paper is the first version, LSSP01000000.
